# Modeling Basins of Attraction for Breast Cancer Using Hopfield Networks

**DOI:** 10.3389/fgene.2020.00314

**Published:** 2020-04-07

**Authors:** Alessandra Jordano Conforte, Leon Alves, Flávio Codeço Coelho, Nicolas Carels, Fabrício Alves Barbosa da Silva

**Affiliations:** ^1^Laboratory of Biological Systems Modeling, Center for Technological Development in Health, Oswaldo Cruz Foundation, Rio de Janeiro, Brazil; ^2^Laboratory of Computational Modeling of Biological Systems, Scientific Computing Program, Oswaldo Cruz Foundation, Rio de Janeiro, Brazil; ^3^Applied Math School, Getúlio Vargas Foundation, Oswaldo Cruz Foundation, Rio de Janeiro, Brazil; ^4^Applied Math School, Getúlio Vargas Foundation, Rio de Janeiro, Brazil

**Keywords:** breast cancer, Hopfield network, basin region of attraction of a minimizer, systems biology, dynamic system

## Abstract

Cancer is a genetic disease for which traditional treatments cause harmful side effects. After two decades of genomics technological breakthroughs, personalized medicine is being used to improve treatment outcomes and mitigate side effects. In mathematical modeling, it has been proposed that cancer matches an attractor in Waddington's epigenetic landscape. The use of Hopfield networks is an attractive modeling approach because it requires neither previous biological knowledge about protein-protein interactions nor kinetic parameters. In this report, Hopfield network modeling was used to analyze bulk RNA-Seq data of paired breast tumor and control samples from 70 patients. We characterized the control and tumor attractors with respect to their size and potential energy and correlated the Euclidean distances between the tumor samples and the control attractor with their corresponding clinical data. In addition, we developed a protocol that outlines the key genes involved in tumor state stability. We found that the tumor basin of attraction is larger than that of the control and that tumor samples are associated with a more substantial negative energy than control samples, which is in agreement with previous reports. Moreover, we found a negative correlation between the Euclidean distances from tumor samples to the control attractor and patient overall survival. The ascending order of each node's density in the weight matrix and the descending order of the number of patients that have the target active only in the tumor sample were the parameters that withdrew more tumor samples from the tumor basin of attraction with fewer gene inhibitions. The combinations of therapeutic targets were specific to each patient. We performed an initial validation through simulation of trastuzumab treatment effects in HER2+ breast cancer samples. For that, we built an energy landscape composed of single-cell and bulk RNA-Seq data from trastuzumab-treated and non-treated HER2+ samples. The trajectory from the non-treated bulk sample toward the treated bulk sample was inferred through the perturbation of differentially expressed genes between these samples. Among them, we characterized key genes involved in the trastuzumab response according to the literature.

## 1. Introduction

Cancer may be caused by genetic and epigenetic factors that deregulate cellular homeostasis. Hanahan and Weinberg (2011) classified cancer deregulated processes in terms of ten hallmarks, which include unlimited proliferative potential, cell death evasion, and angiogenesis, among others.

This disease was described as a pre-existing attractor in Waddington's epigenetic landscape in 2009 by Huang et al. (2009). An attractor is defined as a stable cell state of minimum energy and is associated with a cell phenotype. Due to the stochastic behavior of gene regulation, the attractor is surrounded by a basin of attraction resulting from other gene expression profiles (states) that sustain the same phenotype. The authors proposed that a cancer attractor would be enclosed within epigenetic barriers that should prevent its access. As a cell accumulates mutations and gene deregulation, these epigenetic barriers are lost, and the cancer attractor becomes accessible. This indicates that the epigenetic landscape is not rigid and may change over time (Ao et al., 2008; Huang et al., 2009). In this context, Ao et al. (2008) proposed that cancer can be classified as preventable, curable or incurable, according to its respective functional landscape.

Cancer was also described as an intrinsic robust state of the endogenous network (Ao et al., 2008; Su et al., 2017; Yuan et al., 2017b,c). The endogenous network theory (ENT) is a realistic network dynamic approach, in which the molecular-cellular network is composed of oncogenes, tumor suppressors, and other related agents, and covers most molecular functions. It represents a non-linear stochastic dynamical system able to generate multiple stable states and paths between them (Ao et al., 2008). In this context, coarse-grained modeling was applied using the non-linear Hill functions. The attractors found by this strategy matched gene expression profiles of cell phenotypes from colorectal, prostate, hepatocellular, and gastric cancer. This approach was also applied to acute promyelocytic leukemia and myelopoiesis (Su et al., 2017; Yuan et al., 2017b,c).

The most used methods in gene regulatory networks (GRNs) modeling are stochastic differential equations (SDE), ordinary differential equations (ODEs), and Boolean networks. SDEs have been used to identify the appropriate therapeutic approach against cancer, following the concept of ENT (Su et al., 2017; Yuan et al., 2017b,c). For instance, Yuan et al. (2017b) proposed perturbations that would lead colorectal cancer phenotypes toward the normal intestine phenotype. Either ODEs or SDEs have been used to model the regulation of p53 by MDM2 and MDMX (Leenders and Tuszynski, 2013), the tamoxifen-induced apoptosis in breast cancer (Rouhimoghadam et al., 2018), to predict the impact of combined therapies on myeloma growth (Ji et al., 2016), and to quantify the landscape for cell differentiation and cancer development (Li and Wang, 2013). On the other hand, Cornelius et al. (2013) used differential equations derived from a Boolean network to understand how a leukemia GRN could be switched from an active cell proliferation to an active cell death state. These methods required previous biological knowledge about protein-protein interactions and/or kinetic parameter rates, which may limit the network size and requires an extensive literature search.

The Hopfield network modeling is an alternative method that does not require kinetic parameter rates or protein-protein interactions knowledge. It uses the gene expression profile as input, and the GRN size is only limited by the available computational capacity. This method is a form of a recurrent artificial neural network and was popularized in 1982 by Hopfield (1982). It considers symmetric and asymmetric connections and ensures that sample states converge toward stored attractor patterns during computational modeling.

This method has been used to elucidate cell and cancer development. For instance, Fard et al. (2016) and Guo and Zheng (2017) analyzed single-cell data and identified attractors in the Waddington's epigenetic landscape related to developmental trajectories. In cancer-related reports, Hopfield networks have been used to identify attractors associated with cancer subtypes (Maetschke and Ragan, 2014) and stages (Taherian Fard and Ragan, 2017). Moreover, Szedlak et al. (2014) have used asymmetric Hopfield networks to test densely connected nodes as therapeutic targets and inferred the minimum number of genes necessary for treatment. Meanwhile, Cantini and Caselle (2019) developed a methodology to identify molecular similarities to stratify cancer patients and improve their therapies.

Stratification of patients may improve treatment outcomes through the identification of molecular targets common to a group of patients (He et al., 2019). For instance, trastuzumab is a monoclonal antibody, used as adjuvant treatment against breast and stomach cancers that overexpress the HER2 protein (Wang et al., 2019). Also, triple-negative breast cancer patients may present resistance to neoadjuvant chemotherapy due to pre-existing resistant cell phenotypes (Kim et al., 2018). Both studies were performed considering single-cell sequencing of tumor samples aiming to identify gene expression signatures.

Most one-size-fits-all medicine approach may cause harmful side effects due to low selectivity that might affect both tumor and healthy cells (Siegel et al., 2012). In contrast, personalized medicine considers the tumor of a patient as unique, and identifies genes differentially expressed in tumors in comparison to the surrounding tissue (stroma), which is used as a control (Carels et al., 2015; Conforte et al., 2019). For this reason, personalized medicine is expected to mitigate the side effects and improve treatment efficacy. *In vitro* validation of this approach showed that simultaneous inhibition of target combinations exhibited a more substantial disruptive effect on malignant cells than the sum of single inhibitions (Tilli et al., 2016).

In this report, we identified differentially expressed genes between tumors and their control paired samples from breast cancer patients and used them in Hopfield network modeling. After the characterization of tumor and control attractors, we developed a protocol to identify the best target combination, for each patient, that would minimize potential side effects and withdraw tumor samples from their basin of attraction. For this purpose, we prioritized gene selection according to four criteria: density, node degree, association with cancer-related biological processes, and rate of gene activation in tumor samples. We also performed a further validation of our approach by simulating trastuzumab treatment effects. For that, we used single-cell and bulk RNA-Seq data from three HER2+ breast cancer samples, one treated and two untreated with trastuzumab.

To our knowledge, this is the first report that combines single-cell and bulk RNA-Seq data, personalized treatment concepts, and Hopfield network modeling with the aim of disrupting tumor sample stability.

## 2. Materials and Methods

### 2.1. Identification and Characterization of Differentially Expressed Genes

Bulk RNA-Seq data was obtained from The Cancer Genome Atlas (TCGA) project housed by the Genomic Data Commons (GDC, portal.gdc.cancer.gov), accessed in June 2019. This data set comprises paired tumor and control (stroma) samples from 70 breast cancer patients. We used the FPKM version (Trapnell et al., 2010) normalized by the upper quartile method (Hyndman and Fan, 1996).

scRNA-Seq data, accession number GSE 75688, was obtained in the NCBI Gene Expression Omnibus database, accessed in February 2020. This data set comprises single-cell and pooled samples (bulk RNA-Seq) of primary breast tumor tissue from three HER2+ patients. Among them, one received adjuvant trastuzumab treatment and had 75 RNA-Seq samples available, while two patients did not receive any treatment and had 48 and 18 RNA-Seq samples, respectively (Chung et al., 2017).

We analyzed both RNA-Seq data sets by fold change (FC), aiming to identify differentially expressed genes (DEGs). This method quantifies the change between an initial and final value as the ratio of the final value over the initial one. For the RNA-Seq obtained from TCGA, we considered the tumor expression data as the final value and the control expression data as the initial value. Consequently, positive logFC values indicated higher expression values in tumor samples, while negative logFC values indicated higher expression values in control samples. The logFC values of all genes were calculated individually considering the paired samples of each patient, and then we calculated the average of each gene among all patients. On the other hand, for the RNA-Seq data obtained from NCBI Gene Expression Omnibus, we considered the treated expression data as the final value and the non-treated expression data as the initial value. Consequently, positive logFC values indicated higher expression values in treated samples, while negative logFC values indicated higher expression values in non-treated samples. The logFC values of all genes were calculated considering the bulk RNA-Seq samples because it represents a weighted average of the heterogeneous cells present in the sample.

For both RNA-Seq data sets, we used a *p*-value ≤ 0.01 and a false discovery rate (FDR) ≤ 0.01 as a threshold to select the DEGs. This threshold was associated with an average logFC >3 or < −3.

The DEGs found were characterized using the Gene List tool from the Panther Classification System (pantherdb.org) with respect to their biological process categories, following the Gene Ontology (GO) classification (Thomas et al., 2003; Mi et al., 2010) (Supplementary Table 1).

### 2.2. Clinical Data

We obtained the clinical data of each patient from the TCGA data set in TCGA-GDC, accessed in June 2019 (Supplementary Table 2), and appended data of molecular subtype, entropy, and overall survival. The molecular subtypes were defined according to the classification of The Cancer Genome Atlas Network (2012); the entropy values were obtained from Supplementary File 5 of Conforte et al. (2019); the overall survival (OS) of each patient were determined based on the OS data available in Liu et al. (2018). The OS data was analyzed with the Kaplan-Meier curve using GraphPad Prism software, where 1 is indicative of death, and 0 is indicative of censored data. The resulting curve indicates the percentage of patients alive after the OS time. We considered the overall survival of each patient as the percentage of patients alive on his/her OS time.

### 2.3. Hopfield Network

We applied the discrete neural Hopfield network to perform our analysis. This method implements an auto-associative network that can recover a pattern from partial discrete information (Hopfield, 1982). As input vectors, we used the binarized gene expression profile of each sample. For this, we considered the normal distributions of the logarithm of expression values from DEGs identified for each RNA-Seq data set and each condition (tumor/control or treated/non-treated) separately. We used the geometric mean as the threshold to binarize the gene state in each sample expression profile (Limpert et al., 2001) (Supplementary Table 3). This method allows the identification of genes with different states between samples, which is expected from DEGs. More importantly, it also allows for the identification of genes with the same state between samples, which must be considered since we selected DEGs based on the average logFC value of each gene among all samples from each data set.

The attractors were characterized as the centroids of their respective samples. Each centroid was composed of the average of states for each gene among its samples. The gene state was assigned a value of 1 for an average value > 0.5, and 0 otherwise. Since we used the samples to define each attractor, our method for energy surface construction is parametric. Contrary to the non-parametric method used in Taherian Fard and Ragan (2017), the one applied in this work ensures the existence of basins of attraction related to each attractor.

The Hopfield network analysis was performed with Neupy, a library for neural networks in Python (www.neupy.com). Each attractor from the analyzed data set was used in the training phase to define its weight matrix. The weight matrix (*W*_*a*_) is defined in Equation (1), where *P* is the attractor's gene expression profile, *P*^T^ is its transpose, and *I* is the identity matrix necessary to impose symmetric behavior with diagonal equal to zero. Since *W* may be composed of more than one stored pattern, its value is equal to the sum of all weight matrices (Equation 2).

(1) $$\begin{array}{l} W_{a}=\left(PP^{\text{T}}\right)-I \end{array}$$

(2) $$\begin{array}{l} W=W_{a1}+W_{a2} \end{array}$$

The dynamic trajectory of each sample (*P*_(*t*+1)_, defined in Equation 3), was predicted by following the synchronous approach as shown by Equation (3), where *W* is the final weight matrix, and *P*_(*t*)_ is the sample gene expression profile at time *t*. The *sgn*(*x*) function (Equation 4) determines the binarized output pattern.

(3) $$\begin{array}{l} P_{\left(t+1\right)}=sgn\left(P_{\left(t\right)}W\right) \end{array}$$

(4) $$\begin{array}{l} sgn(x)\text{ =}\{\begin{array}{ll} \text{ 1} & :x\geq \text{0}\\ \text{ 0} & :x<\text{0} \end{array} \end{array}$$

By analogy to a physical system, the discrete Hopfield network energy (*E*) is calculated using the Lyapunov function, which guarantees convergence to a low-energy attractor state (see Equation 5) (Taherian Fard and Ragan, 2017).

(5) $$\begin{array}{l} E\left[P\left(s\right)\right]=\frac{-1}{2}PWP^{\text{T}} \end{array}$$

where *E*[*P*(*s*)] is the energy of network state *s* for sample vector *P* and time *t*.

### 2.4. Samples Characterization

For the RNA-Seq data set from TCGA, the Euclidean distances (EDs) were calculated based on all network dimensions and implemented following three strategies: (i) calculation of the EDs between each sample and all other samples that converged to the same attractor, whose respective average was used to infer the sizes of the basins of attraction for the tumor and control sample attractors; (ii) calculation of the EDs between tumor samples and the control attractor (centroid); and (iii) calculation of the EDs between tumor samples and the tumor attractor (centroid). These values were correlated with the patients' clinical data.

We set two conditions to ensure the statistical significance of data correlations despite the data heterogeneity. First, the data of each clinical variable (tumor stage, molecular subtype, entropy, and overall survival) should group into at least three classes. Second, each class should include at least three patients to infer the average of the respective class.

The non-parametric Kruskal-Wallis test (Kruskal and Wallis, 1952) and the pairwise Wilcoxon signed-rank test (Wilcoxon, 1945) were performed to evaluate if all classes of the same clinical variable were significantly different. The null hypothesis of the non-parametric Kruskal-Wallis test is that all classes have the same average ED. When the null hypothesis was rejected, a pairwise Wilcoxon signed-rank test was performed to identify which class significantly deviated from the average. This test was performed for tumor stage, molecular subtype, and entropy clinical variables. For overall survival, we performed the Pearson correlation test. These statistical analyses were performed in R.

For the RNA-Seq data set from NCBI Gene Expression Omnibus database, we characterized the samples using principal component analysis (PCA) and a t-distributed stochastic neighbor embedding analysis (t-SNE) (Maaten and Hinton, 2008). Both tests were performed in Python.

### 2.5. Target Identification

Each DEG found for paired tumor and control samples was classified according to four parameters. For each parameter, the gene priority was screened in ascending and descending order. Parameter 1: density of each gene in the Hopfield network; Parameter 2: number of GOs related to cancer development associated with each of the DEGs; Parameter 3: number of patients with the gene under consideration active (1) in their tumor samples (biomarker); and Parameter 4: node degree of each gene.

Parameter 1 was determined following Equation (6), where the density (*D*) of node *i* is the sum of all weights in *W* for node *i* divided by the number of network nodes (*n*). Negative values for *w*_*ij*_ indicated different states for nodes *i* and *j* in the stored patterns, while the opposite is true for positive values.

(6) $$\begin{array}{l} D_{i}=\frac{1}{n}\overset{j=n}{\underset{j=1}{\sum }}w_{ij} \end{array}$$

The second parameter is determined by the number of GOs, identified in the Panther Classification System as related to cancer development, associated with each DEG. On the other hand, parameter 3 identified the number of patients with an active DEG in the tumor sample and inactive in it respective control sample. In this case, we may hypothesize that genes active in many tumor samples, and inactive in their respective control samples, may be considered as breast cancer biomarkers.

The node degree of each gene, parameter 4, was determined according to the human interactome, obtained from the intactmicluster.txt file (version updated December 2017) accessed on January 11, 2018, at ftp://ftp.ebi.ac.uk/pub/databases/intact/current/psimitab/intact-micluster.txt. This file presents 151,631 interactions among 15,526 human proteins with UniProtKB accessions. The node degree of each protein was calculated through automated counting of their edges (Supplementary Table 4). We analyzed the node degrees of DEGs for which we found equivalence between the Ensemble and UniProtKB accessions (215/324), used for the RNA-Seq data and the interactome, respectively.

As stated in algorithm 1, we tested the effect of switching off 1–20 genes, according to priority lists, and analyzed the resulting energy values. This experiment was performed with the aim of identifying the number of genes that needed to be inhibited to move tumor samples away from their tumor basin of attraction. Our strategy followed the personalized medicine concept, considering the paired tumor and control samples of each patient. Moreover, to avoid potential side effects, we only switched off genes that were active (1) in the patient's tumor sample and inactive (0) in the paired control sample (stroma).

The implementation of algorithm 1 is available upon request. The algorithm considers two functions: **length**, which is the vector extent, and **energy**, which calculates the sample-related energy as described above. We analyzed each patient's tumor (*patientTumorSample*) and control (*patientControlSample*) sample gene expression profiles and searched for genes (*gene*) in the gene priority lists (*listOfGenes*). If a gene was active in the patient tumor sample and inactive in its paired control, it would be inhibited, and a new patient gene expression profile (*patientTreated*) was retrieved. If the change was not sufficient to reach an energy level equivalent to the barrier height between the two attractors (−35,000), the algorithm would continue to the second gene (*gene*) in the gene priority list (*listOfGenes*). When the barrier height energy was reached, the algorithm would leave the “for” loop, and the next patient gene expression profile would be analyzed. This algorithm returns the number of inhibitions indicated to move each tumor sample away from the tumor basin of attraction.

**Algorithm 1:** 

1: *Input*: Priority list of genes for inhibition (*listOfGenes*) / Patient control sample expression profiles (*patientControlSample*) / Patient tumor sample expression profiles (*patientTumorSample*).
2: *Output*: Combination and number of gene inhibitions recommended for each patient (*inhibitedGenes*, length of *inhibitedGenes*).
3: **procedure** Gene inhibition(*listOfGenes*, *patientControlSample*, *patientTumorSample*)
4: *Energy* = 0
5: *attemps* = 0
6: *patientTreated* = *patientTumorSample*
7: *inhibitedGenes* = {}
8: **while** (length (*inhibitedGenes*) <20) and (*attemps* <100) **do**
9: *attemps* += 1
10: **for** **all** *gene* in *listOfGenes* **do**
11: **if** *gene* = 0 in *patientControlSample* **then**
12: **if** *gene* = 1 in *patientTumorSample* **then**
13: *gene* = 0 in *patientTreated*
14: **add** *gene* to *InhibitedGenes*
15: **if** energy(*patientTreated*) ≥−35000 **then**
16: **break**
17: **return** inhibitedGenes, *length(inhibitedGenes)*

A similar algorithm was used to test the gene target combinations able to move tumor samples toward the control attractor. In this case, we tested switching off 1–50 genes, according to the priority lists. Instead of calculating the energy of the new patient gene expression profile (*patientTreated*) in line 15 of algorithm 1, we predicted its convergence toward the tumor or control attractor. If the changes were not sufficient to induce tumor sample convergence toward the control attractor, then the algorithm would continue to the next gene (*gene*) in the gene priority list (*listOfGenes*); otherwise, it would leave the “for” loop, and the next patient gene expression profile would be analyzed. In this case, the algorithm returns the number of inhibitions indicated to move each tumor sample toward the control attractor.

For trastuzumab treated and non-treated patients, we simulated the effect of trastuzumab treatment in the non-treated RNA-Seq sample, aiming to further validate our personalized approach. To do this, we built an energy landscape with single-cell and bulk RNA-Seq samples from non-treated patient 1 and the treated patient. We could not perform this experiment for non-treated patient 2 because it did not have enough samples to build its basin of attraction.

The state transition from the non-treated bulk RNA-Seq sample toward the treated bulk RNA-Seq sample was inferred through the perturbation of genes differentially expressed between those samples. Each DEG would receive the same state than the one in the treated bulk RNA-Seq sample, creating a new transitory state. The connection between all transitory states defines the trajectory from the non-treated basin of attraction toward the treated one. Besides, we characterized each DEG according to their role in signaling pathways associated with trastuzumab response (Supplementary Table 7).

We used bulk RNA-Seq samples as reference in the state trajectory because they comprise single-cell heterogeneity and abundance, as a weighted average of all single-cell samples available.

## 3. Results

### 3.1. Characterization of Differentially Expressed Genes

**Figure 1:**
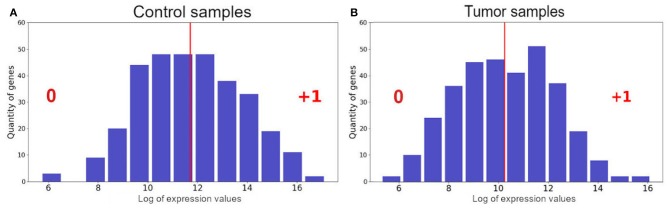
Distribution of the logarithm of expression values from control samples **(A)** and tumor samples **(B)**. The red line represents the geometric mean. The numbers “0” and “+1” are the binarized states that each gene received according to each sample expression profile.

We identified 324 DEGs among the paired tumor and control samples. We binarized the gene expression profiles from each patient following the normal distribution found for the logarithms of expression values (RNA-Seq data) of all tumor and control samples, separately, then used the geometric mean as a threshold (Limpert et al., 2001) (Figure 1). It is important to highlight that the results are sensitive to the chosen threshold and the geometric mean is the best fit for our samples (Supplementary Table 3).

From the 324 DEGs, 295 were recognized by the Panther Classification System. The Gene List tool found 1,918 GO codes related to biological processes, among which 111 were related to cancer development or response. Of all DEGs, 65.4% were classified with at least one cancer-related GO. All of these results can be analyzed in more detail using Supplementary Table 1.

**Figure 2:**
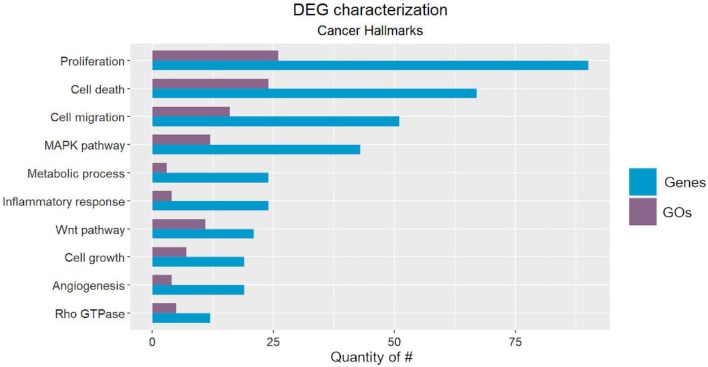
Number of Gene Ontology (GO) codes found for each cancer-related hallmark/pathway (purple) and number of Differentially Expressed Genes (DEGs) classified according to each GO (blue).

We classified the cancer-related processes into ten onco- or tumor suppressor processes (Figure 2), from which seven corresponded to well-described hallmarks, and three were cancer-related pathways. The identified hallmarks were proliferation, cell death, cell migration, metabolic process, inflammatory response, cell growth, and angiogenesis, while the cancer-related pathways were the MAPK, WNT, and Rho GTPase pathways (Hanahan and Weinberg, 2011).

Note that inflammatory-related GOs could not be differentiated into acute or chronic response, which hampers elucidation of association with cancer formation, since it can only be triggered by chronic inflammation (Gonzalez et al., 2018). Furthermore, we included the regulation of mTOR signaling in both the proliferation and cell death categories due to its essential role in these hallmarks (Tian et al., 2019). Lastly, the hypoxia response characterized the metabolic cancer hallmark and is triggered by the hypoxia-inducible factor (HIF), which is directly related to the establishment of the “Warburg Effect.” This metabolic rewiring occurs when tumor cells activate ATP generation via glycolysis (Simon, 2006; Liberti and Locasale, 2016). This process is essential for cancer cell survival under hypoxic stress. HIF transcriptionally regulates hundreds of genes that are also related to invasion, metastasis, genetic instability, and immune response (Yan et al., 2019).

Among the pathways that we identified, the MAPK pathway has been characterized as a key regulator of cancer development and is associated with several cellular processes, such as proliferation, growth, apoptosis, and migration. It involves other essential kinases (ERK and JNK) and proteins (RAS, Raf, and MEK), which can be reviewed in more detail in Dhillon et al. (2007).

The WNT pathway is divided into two main types: canonical and non-canonical (via JNK cascade) signaling pathways. Its function was first described in the developmental processes of *Drosophila melanogaster* and has been recently associated with cancer cell proliferation, stemness, metastasis, and immune evasion. This pathway can be reviewed in Zhan et al. (2017).

Finally, the Rho GTPase pathway has been associated with remodeling of the actin cytoskeleton, which is related to cell division and phenotype transition. It participates in cancer cell migration, proliferation, survival, and death. Its role in all of these signaling pathways can be reviewed in more detail in Haga and Ridley (2016).

### 3.2. Attractors Analysis

The Euclidean distances (EDs) were calculated considering all data dimensions. The EDs between all tumor and control paired samples revealed that the tumor basin of attraction was, indeed, larger than that of the control, when considering all samples that converged to the expected attractor. However, the size difference between both basins of attraction, considering samples that converged to each attractor, depended on the second decimal value and may not be considered as meaningful (Table 1). The difference among ED considering all samples and ED considering samples that converged to each attractor can be explained by the fact that five tumor samples converged to the control attractor, and because of that, were included in the ED measurement of the control basin of attraction. Interestingly, these samples were classified by (i) molecular subtypes with good prognosis (LumA or LumB); (ii) being in the initial stages of tumor development (stages i and iib); and (iii) most of them (A0BM, A0C3, A1EU, and A2FF) presenting the smallest entropies among patients (Supplementary Table 2), which is associated with a low level of aggressiveness (Breitkreutz et al., 2012; Conforte et al., 2019).

**Table 1:** Tumor and control basin of attraction sizes according to the Euclidean distances of all samples and samples that converged to each attractor.

	**ED for all samples**	**ED for samples that converged to each attractor**
Tumor	8.34	8.19
Control	7.92	8.17

**Figure 3:**
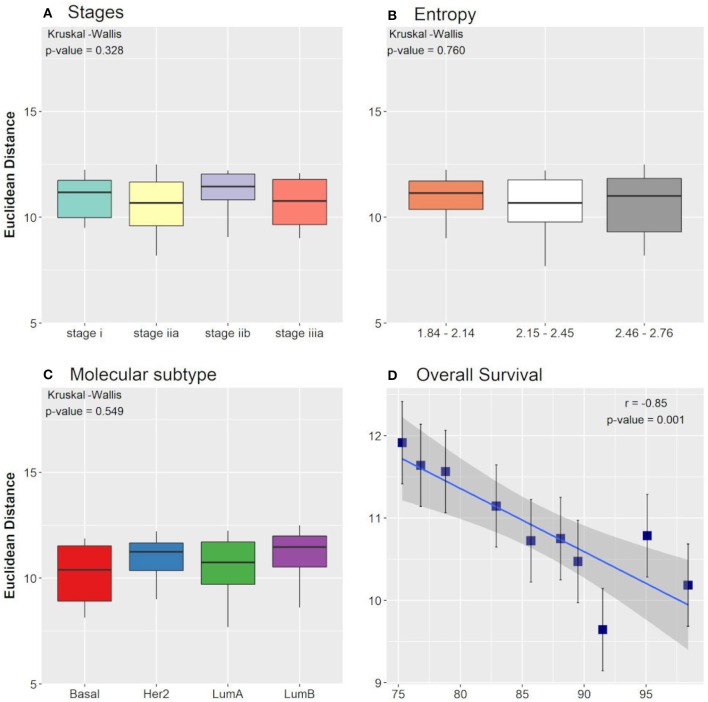
Boxplot of Euclidean distances found between the control attractor and tumor samples, considering cancer stage **(A)**, entropy **(B)**, and molecular subtype **(C)** clinical data classifications. The *p*-values found in Kruskal-Wallis test are indicated in each figure. **(D)** Pearson correlation between the overall survival clinical data classification and the Euclidean distances found between the control attractor and tumor samples. The correlation coefficient (r) and the *p*-value found are indicated in the figure.

When comparing the clinical data with the EDs between the control attractor and the tumor samples that converged toward the tumor attractor, we found that the averages among the groups of tumor stages, molecular subtypes, and entropies were not significantly different when considering the non-parametric Kruskal-Wallis test. The result is the same when considering the ED between the tumor attractor and the tumor samples that converged toward the tumor attractor. Nevertheless, the Pearson correlation test, performed for the overall survival groups, showed a significant negative correlation with the EDs between the control attractor and the tumor samples that converged toward the tumor attractor (*r* = −0.85). A *p*-value = 0.001 and a slope = −0.07, with a 95% confidence interval between −0.11 and −0.03, which indicates that the regression line slope is different than zero. As expected, smaller distances are related to higher overall survival. No correlation was found among the overall survival groups considering the ED between the tumor attractor and the tumor samples that converged toward the tumor attractor. The Kruskal-Wallis test and the Pearson correlation test results are shown in Figure 3.

**Figure 4:**
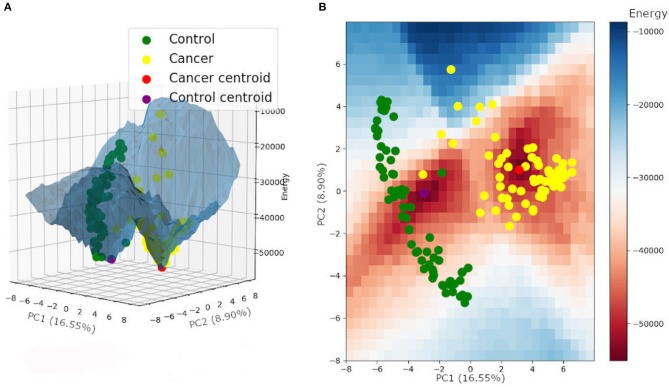
Energy landscape built for control and tumor attractors, and samples, plotted on a three-dimensional **(A)** and a two-dimensional grid **(B)**.

The same energy value was found for both tumor and control attractors (−55,000), corresponding to the local minimum of the energy function. The potential energy analysis revealed that tumor samples are more associated with a lower energy level and are closer to their attractor minimum energy than the control samples (Table 2) (for more details, see Supplementary Table 5). This result follows a common biological trend of tumors presenting alternative pathways that ensure tumor stability and promote tumor resistance to chemotherapy. The energy landscape based on those samples is presented in Figure 4.

**Table 2:** Average energy, average energy distance between samples and their respective attractor energy minimum and attractor energy minimum for tumor and control samples.

	**Average energy of samples**	**Average energy distance between samples and attractors**	**Minimum (attractor) energy**
Tumor	−31,338	24,188	−55,000
Control	−29,426	26,100	−55,000

### 3.3. Attractor Transitions

Hypothesizing that the genes involved in tumor state stability are essential for tumor maintenance, it may be reasonable to argue that the disruption of their products (mRNA, protein) might lead to tumors moving toward an attractor associated with active cell death. Accordingly, we searched for the best key gene combinations for each patient by considering their gene expression profiles that, when switched off, would minimize side effects and move tumor samples away from the tumor basin of attraction. This exercise provides a measure of how many key genes should be inhibited in each tumor sample to withdraw it from the tumor basin of attraction.

Supplementary Table 6 presents the values attributed to each gene when considering the four prioritization parameters: density of each gene in the Hopfield network; number of GOs related to cancer development associated with each gene; number of patients with the gene under consideration active (1) only in their tumor samples (biomarker); and node degree. Each parameter was analyzed in ascending and descending order.

**Figure 5:**
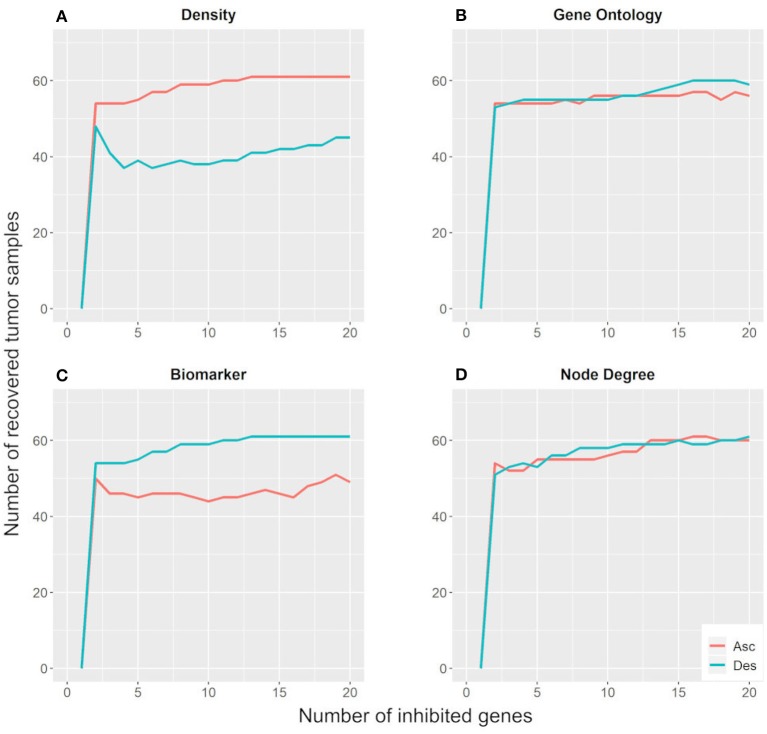
Number of tumor samples that moved away from the tumor basin of attraction, according to the number of genes inhibited for each parameter: density **(A)**, gene ontology **(B)**, biomarker **(C)**, and node degree **(D)**. “Asc” represents the ascending order, while “Des” represents the descending order.

Figure 5 shows that the inhibition of two targets would be sufficient to move ~55 tumor samples (78.6%) away from their basin of attraction. Moreover, the parameters regarding the number of GOs and node degree were not effective for identifying key genes with the potential to change gene expression patterns in the Hopfield network since their ascending and descending orders exhibit similar behavior. On the other hand, the biomarker and density parameters showed different behaviors when considering their ascending and descending orders. The descending biomarker curve moved more tumor samples away from their basin of attraction than its respective ascending curve, while the opposite trend was observed for the node density parameter. These curves are similar due to their node selection strategy.

The descending biomarker curve prioritizes genes that are active in several tumor samples and inactive in their respective paired control samples. In other words, these genes present different states between tumor and control samples for most patients. Similarly, the ascending density curve prioritizes nodes with negative connection weights in the weight matrix. As revealed in the methodology section, interacting nodes with different states present a negative connection weight. Additionally, we impose the restriction that only nodes active in tumor samples and inactive in control samples are suitable for inhibition. For these reasons, the descending biomarker order and the ascending density order of prioritization matched.

According to the biomarker classification, CNTFR-alpha presented the highest value (70), which means that it was active in all tumor samples analyzed and inactive in all normal samples. CNTFR-alpha has been associated with proliferation and poor prognosis and been proposed as a biomarker of low-grade gliomas (Lu et al., 2012; Fan et al., 2017). SGK2 and PLP1 appeared in the second position of biomarker classification, being active in the tumor samples of 69 patients and inactive in their respective controls. SGK2 has been associated with hepatocarcinoma progression and bladder cancer cell proliferation, migration, and invasion (Liu et al., 2017; Chen et al., 2018). PLP1, although active in most tumor samples analyzed in this work, has been recently described as consistently downregulated in several cancer types, including breast cancer (Li et al., 2017). We did not find any lines of evidence in the literature that enable its association with cancer development. Nevertheless, it is described as a cancer gene in GeneCards, and its antibody is an effective inhibitor of cell growth in breast cancer (www.mybiosource.com—#7~005540).

**Figure 6:**
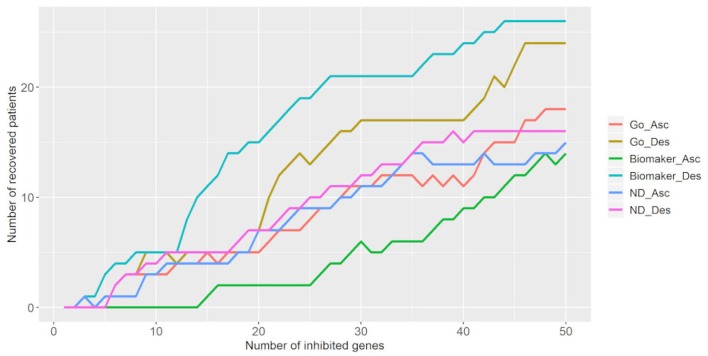
Number of tumor samples that converged toward the control attractor, according to the number of genes inhibited for each parameter: gene ontology (GO), biomarker, and node degree (ND). “Asc” represents the ascending order, while “Des” represents the descending order. The biomarker ascending curve matches the density descending curve, and the biomarker descending curve matches the density ascending curve.

We also tested the number of targets necessary to bring tumor samples toward the control attractor. Figure 6 shows that the inhibition of 50 targets is necessary to bring 18–26 tumor samples (25.7–37.2%) to the control attractor. As stated above, the descending biomarker curve and ascending density curve were the most promising gene selection parameters since they enabled movement of the largest number of tumor samples toward the control attractor, through the inhibition of few genes. This result indicates that it is not feasible to bring a tumor sample back to the normal phenotype.

### 3.4. Simulation of Trastuzumab Treatment Effect

**Figure 7:**
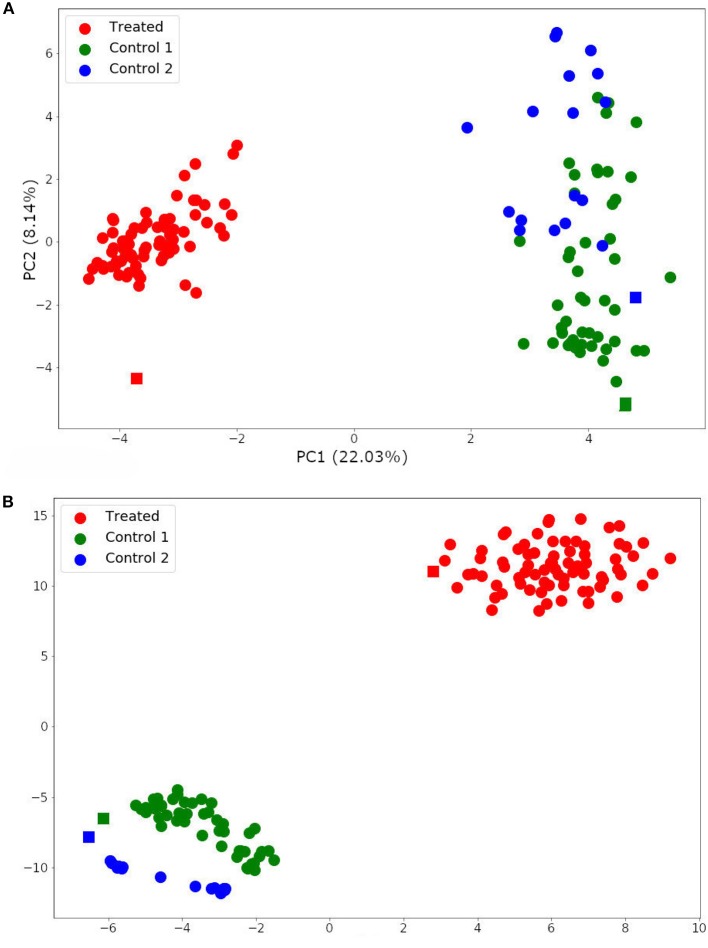
Single-cell and bulk RNA-Seq data analysis by Principal Component Analysis (PCA) **(A)** and t-distributed stochastic neighbor embedding (t-SNE) **(B)**. Spheres represent single-cell samples and squares represent their respective bulk RNA-Seq samples.

The two-dimensionality reduction methods, PCA and t-SNE, were able to separate gene expression profiles from treated and non-treated single-cell and bulk samples according to their similarities. The PCA analysis is plotted in Figure 7A. This figure shows that the PCA was able to explain the variance among the samples with two principal components (PCs), and separated treated and non-treated samples into two clusters. The first PC explained 22.03% of the total variance, while the second PC explained 8.14% of it.

The t-SNE analysis (Figure 7B) also separated the samples into two main clusters: trastuzumab treated and non-treated samples. Moreover, it distinguished non-treated samples of each HER2+ patient. These results indicated that the DEG selected for this data set not only represents the trastuzumab treatment but also, the differences between the gene expression profile from HER2+ patients. In addition, these results are in agreement with previously published results (Wang et al., 2019).

**Figure 8:**
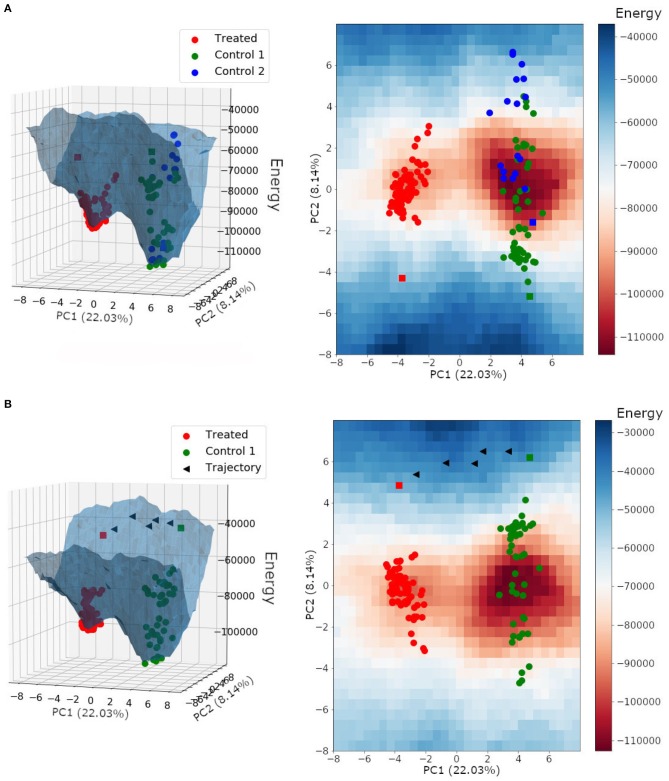
**(A)** Energy landscape for HER2+ breast cancer samples plotted on a three-dimensional grid and a two-dimensional grid. **(B)** Energy landscape plotted on a three-dimensional grid and a two-dimensional grid, with the trajectory between the non-treated bulk RNA-Seq sample from patient 1 (control 1) and the treated bulk RNA-Seq sample (treated). Each triangle represents the perturbation of a subset with ~30 DEGs. Spheres represent single-cell samples and squares represent their respective bulk RNA-Seq sample.

The energy landscape built for HER2+ treated and non-treated samples is shown in Figure 8A. It is interesting to note that samples from both non-treated patients shared the same basin of attraction, even though we performed the Hopfield training phase considering three attractors, one for each patient. Besides, the trastuzumab treated samples composed a different basin of attraction with a minimum energy higher than the one found in the non-treated basin of attraction. This result indicated that non-treated samples have a higher tumor phenotype stability than treated samples, and agrees with the fact that trastuzumab treatment is, indeed, an adjuvant therapy approach rather than a healing one.

We can also observe in Figure 8 that the bulk RNA-Seq samples (squares) may be away from their respective single-cells (spheres). This may occur because bulk RNA-Seq is a weighted average of all single-cells in the tumor tissue, and because of that, influenced by single-cell relative amounts. Besides, the bulk RNA-Seq may comprise single-cell phenotypes that were not obtained during sequencing.

Paired samples from the same patient, before and after trastuzumab treatment, were not available. We used samples from three different patients, two non-treated with trastuzumab (patients 1 and 2) and one treated (patient 3). Since samples from patient 1 and 2 belonged to the same region of the epigenetic landscape (see Figure 8), we hypothesized that their corresponding treated samples would also be in the same basin of attraction composed of samples from patient 3.

In this context, we tested the effects of trastuzumab treatment in the non-treated bulk RNA-Seq from patient 1. To do this, we built an energy landscape based on non-treated patient 1 samples and the treated patient samples. We identified 172 differentially expressed genes between the non-treated and treated bulk RNA-Seq samples. All DEGs were perturbed according to the treated gene expression profile. Those changes created new transitory states that, when connected, formed the trajectory between both basins of attractions (Figure 8B).

The target profile was reached after the perturbation of all 172 DEGs, and each triangle in Figure 8B represents the perturbation of a subset with ~30 DEGs. The trajectory found was mapped in the energy landscape defined by the Hopfield network, and is one among many other possibilities. This trajectory was not optimized because we did not consider the associated signaling pathways, which would reduce the number of DEGs to be perturbed.

This experiment was not performed for the non-treated samples from patient 2 because it did not have enough samples to build its basin of attraction.

Changing 172 genes expression values is not feasible in the medical context, if we consider each intervention individually. However, key genes may initiate a cascade response that involves many others. For this reason, we characterized the DEGs concerning their respective biological processes in the Panther Classification System. Among the 172 DEGs, 92 were characterized by the Panther Classification System with at least one biological process (Supplementary Table 7). We reduced this analysis considering the biological processes involved in the trastuzumab treatment response. Among them, we can highlight cell proliferation, transcription, apoptosis, motility, and immune response (Herbst, 2004; Shi et al., 2014). Through a literature search, we saw that the genes involved in those biological processes have their role, in trastuzumab treatment, characterized.

The STAT1 gene is involved in proliferation and transcription biological processes. This gene plays an important role in HER2 inhibition and is activated after the trastuzumab treatment, through interferon-gamma production by the mobilized natural killer cells (Shi et al., 2014). This interferon-gamma production also activates the HLA-A antigen, involved in the immune response (Chaganty et al., 2015). The MEL-18 gene (or PCGF2) was classified in the transcription biological process. This gene is described as essential for trastuzumab treatment since its inhibition may result in a trastuzumab-resistant phenotype (Lee et al., 2019).

In this context, CCR7, PIP, and GBP1 genes were classified in the immune response biological process. CCR7 determines a cancer stem cell phenotype through the Notch signaling pathway, and PIP belongs to the PI3K signaling pathway. Both signaling pathways are related to trastuzumab treatment resistance (Pohlmann et al., 2009; Baker et al., 2014; Boyle et al., 2017). Besides, GBP1 and IFI27 were associated with the apoptotic biological process, and were related to breast cancer phenotype resistant to trastuzumab treatment (von der Heyde et al., 2015).

New therapeutic targets have been explored to overcome trastuzumab resistance. The ITGB6 gene, associated with motility biological process, has been proposed as a therapeutic target through inhibition by 264RAD antibody. Its combination with trastuzumab treatment was able to stop tumor growth even in trastuzumab-resistant cells (Moore et al., 2014).

The results obtained in this section propose a further validation of our personalized approach.

## 4. Discussion

The Hopfield network was efficient in revealing cancer attractors related to molecular subtypes and developmental stages in previous works (Maetschke and Ragan, 2014; Taherian Fard and Ragan, 2017). In this report, we considered the gene expression profile of paired tumor and control samples from breast cancer patients to analyze both normal and tumor attractors, infer the best target combinations able to withdraw the tumor sample from its basin of attraction and simulate the trastuzumab treatment effect in non-treated bulk RNA-Seq sample.

Among our data, only five tumor samples converged to the control attractor. These samples presented molecular subtypes with a good prognosis, were in initial stages of cancer development, and had low entropy values. The Shannon entropy has been widely explored as a cancer development measure and aggressiveness indicator. Higher entropy values are associated with aggressive tumor phenotypes. This correlation was found when comparing cancer and control cells, advanced and initial stages of tumor development, aggressive cancer types and good prognosis cancer types (Breitkreutz et al., 2012; Winterbach et al., 2013; Banerji et al., 2015; Conforte et al., 2019).

The energy values of tumor samples were closer to the tumor attractor minimum compared to control samples. Although this result differs from the one published by Taherian Fard and Ragan (2017), this behavior is expected because tumor samples have alternative pathways that ensure their phenotypic stability (Fumi and Martins, 2013; Taherian Fard and Ragan, 2017).

Also, the correlation between the Euclidean distances from tumor samples to the control attractor and the patient's overall survival indicates that there is a higher chance of treatment success when the gene expression profile is not yet fully reprogrammed for cancer development. However, those results did not hold considering the Euclidean distance from tumor samples to the tumor attractor. This analysis also revealed that the tumor basin of attraction is larger than that of the control and should comprise more heterogeneous data, as indicated by Taherian Fard and Ragan (2017).

The analysis of single-cell data allowed us to see the tumor basin of attraction in more detail and, along with the results discussed above, indicates that there may be multiple basins of attraction related to cancer development, rather than one large basin of attraction that comprises all cancer samples. Cancer basins of attraction could be composed of similar gene expression profiles. For instance, samples from the same molecular subtype.

In this context, our results showed that non-treated patient 2 did not have enough samples to build its own basin of attraction, but its samples were distributed in the basin of attraction built for the non-treated patient 1. Both were characterized as HER2+ breast cancer molecular subtype. Moreover, the trastuzumab-treated samples composed a new basin of attraction with higher minimum energy than the non-treated one. This result agrees with the trastuzumab adjuvant role in cancer therapy.

The protocol developed for the identification of potential therapeutic targets matches the concept of personalized medicine. Specific target combinations were derived from the gene expression profile of each patient, with the potential to mitigate side effects and enhance the treatment outcome.

Among the parameters tested to indicate gene priority, the node degree has been indicated as a key factor (Carels et al., 2015; Tilli et al., 2016; Conforte et al., 2019). Other topological measures of gene regulatory networks (GRNs) have also been widely used in the identification of new therapeutic targets (Peng and Schork, 2014; Azevedo and Moreira-Filho, 2015). However, they could not be inferred in this work because the Hopfield network approach does not consider protein-protein interactions.

The best parameters, according to our results, were genes with high number of patients that present the node active only in tumor samples and low-density values. Both had the largest potential for tumor sample destabilization with fewer gene inhibitions. The difference between their effect and the effect of its opposite priority order was small. This small effect difference was also observed for other parameters. This may occur because the Hopfield network can be viewed as a highly connected network, which hampers the characterization of each node's impact on the network. Yet, the observations in both cases are biologically coherent.

The Hopfield network is highly connected, but each interaction has its own weight. Consequently, the Hopfield network is heterogeneous, and the weighted interactions allows the differentiation between important and non-important connections. The biological coherence is related to higher effects expected after inhibition of nodes that are active in most tumor samples and inactive in their respective control samples. This indicates that those genes have an essential role in tumor development. For instance, PLP1 was active in most tumor samples and inhibited in the respective control samples. Its antibody is an effective inhibitor of cell growth in breast cancer.

The identification of therapeutic targets was further validated through simulation of the trastuzumab treatment effect in the non-treated bulk RNA-Seq data from patient 1. We determined the trajectory from non-treated to treated basins of attraction for patient 1 and identified key genes involved in the trastuzumab treatment response.

The trastuzumab adjuvant treatment is indicated for HER2+ breast cancer patients, but there are cases of trastuzumab treatment resistance (Han et al., 2019). In this context, the energy landscape obtained for HER2+ samples could determine the state space of gene expression profiles that could be indicated for effective trastuzumab treatment. Also, the trajectory between the treated and the non-treated basins of attraction may indicate new potential therapeutic targets. These could be used in combination with trastuzumab, such as the ITGB6 specific antibody 264RAD, to increase the state space of gene expression profiles with available treatment. This approach could be explored in the context of personalized medicine in future studies.

The Hopfield network succeeded in modeling the basins of attraction for both bulk and single-cell RNA-Seq. This method is entirely based on the gene expression data and considers the differentially expressed genes among our samples, which is essential due to tumor heterogeneity. As an advantage, it does not require a fully-featured network or literature search about protein-protein interactions.

Other modeling methods have also been proposed in order to identify appropriate therapeutic approaches against cancer. For instance, Su et al. (2017) and Yuan et al. (2017b,c) applied the Endogenous Network Theory (ENT) with a coarse-grained modeling, using the non-linear Hill function. They found attractors that matched gene expression profiles of cell phenotypes related to colorectal, prostate, hepatocellular, and gastric cancer (Su et al., 2017; Yuan et al., 2017b,c). In this context, Yuan et al. (2017b) proposed that colorectal cancer could be treated, and reach a normal intestine phenotype, through suppression or promotion of the inflammation program, suppression of retinoic acid signaling, and suppression of anti-inflammation process, according to the cancer cell phenotype. Moreover, the ENT indicates that cancer can be classified as preventable, curable or incurable according to its respective functional landscape.

As proposed by ENT, our results indicated the existence of different functional landscapes for tumor samples. However, the ENT considers the transition from cancer to the normal state as feasible. In this research, we observed that more than 20 gene inhibitions are required to move a tumor sample from the tumor toward the control basin of attraction. This result indicates that recovering a tumor sample back to the control state is not feasible, which may be explained by accumulation of genetic mutations, alteration in genes copy-number, and other processes that may not be regulated by drug administration (Van Bockstal et al., 2020). Rather, we identified the key genes responsible for attractor stability. By extension, one could argue that key genes are essential to tumor biology and that their inhibition would lead to cell death (Tilli et al., 2016).

Biological networks are typically asymmetrical, and several modeling paradigms consider this asymmetry explicitly. For instance, Kwon et al. proposed a stochastic dynamic decomposition method to analyze the dynamics near stable or unstable states. This modeling approach can generate the landscape and the associated energy function, considering the inherent asymmetry of biological networks (Kwon et al., 2005; Yuan et al., 2017a). However, this approach is based on stochastic differential equations, and requires parameters related to each interaction. Those parameters are normally defined by extensive literature search or wet-lab experiments. Furthermore, Li and Wang showed that the potential landscape based on differential equations is susceptible to parameter changes. Nevertheless, stochastic differential equations may offer several advantages, such as a global landscape, reduced sampling space of paths between two states, and relative stability between stable states in the presence of the system's noise (Tang et al., 2017). Besides, Yuan et al. (2017c) presented a non-linear and coupled SDE system that models stable states with relatively large basins of attraction, and showed that this model is insensitive to interaction details at the core network level, by performing random parameter tests. Furthermore, Toulouse et al. (2005) suggested that the attractor robustness to small amounts of noise on SDE models is related to the presence of network motifs.

Asymmetric Hopfield networks do not have a general method to obtain the energy function. Previously published works used symmetric Hopfield network with a non-parametric training approach (Maetschke and Ragan, 2014; Taherian Fard and Ragan, 2017). These authors discussed that the resulting landscape is a gross approximation of the biological reality. We improved this aspect in our work using a parametric training approach, which receives more biological information and limits the number of possible basins of attraction. Consequently, our approach better characterizes the landscape and approximates it to the biological reality. The energy landscape obtained is data-driven and may represent the biological reality without the noise of estimated parameters.

Our approach combined Hopfield networks with the application of personalized medicine, by considering a large subset of data from real patients effectively involved in oncogenesis. Hopfield network modeling was successful in (i) identifying and characterizing both tumor and normal attractors; (ii) associating tumor sample locations, in the epigenetic landscape, with clinical data; (iii) identifying target combinations whose inhibition would be more efficient in moving tumor samples away from their basin of attraction; (iv) simulating the effects of trastuzumab treatment in non-treated bulk RNA-Seq data; and (v) inferring the trajectory between trastuzumab treated and non-treated basins of attraction.

## 5. Conclusion

We used Hopfield network modeling to analyze cancer and control attractors based on real patient data and associated their locations, in the epigenetic landscape, with clinical data. Our results indicate that the larger the Euclidean distance between the tumor sample and the control attractor, the lower the patient overall survival is. Besides, tumor samples' energies imply a stable phenotype that requires a combination of changes, specific to tumor sample, to move them away from its basin of attraction. We developed and applied a protocol to identify the key genes in tumor phenotype stability. Since these key genes are essential for sustaining tumor biology, we suggest that their combined inhibition would be helpful in patient treatment. This protocol followed the personalized medicine concept in its three main aspects: considering each tumor as unique, mitigating harmful side effects, and enhancing the treatment outcome. We further validated our approach by simulating the trastuzumab effect in non-treated RNA-seq data and identifying the trajectory from the non-treated to the treated basin of attraction. The key genes involved in the state transition were characterized according to their biological processes and participation in trastuzumab-related pathways.

## Data Availability Statement

All datasets generated for this study are included in the article/Supplementary Material.

## Author Contributions

AC and LA participated in the conception, design, analysis, and interpretation of the work. FC participated in the conception, design, and drafting of the work. NC participated in the design of the work and substantially revised it. FS participated in the conception, design, analysis, and interpretation of the work and substantially revised it. All authors participated in the report writing and approved the final version.

### Conflict of Interest

The authors declare that the research was conducted in the absence of any commercial or financial relationships that could be construed as a potential conflict of interest.

## Supplementary Material

Supplementary Table 1 Characterization of each DEG identified in the RNA-Seq data set from TCGA concerning their respective biological process, following the Gene Ontology (GO) classification.

Supplementary Table 2 Clinical data, molecular subtype, entropy, and overall survival for each patient from TCGA data set.

Supplementary Table 3 Analysis of sample convergence after threshold variation. The geometric mean best fitted our samples and is indicated as the threshold to binarize the gene state in each sample expression profile.

Supplementary Table 4 Node degree for differentially expressed genes.

Supplementary Table 5 Samples energy and Euclidean distances between samples and attractors.

Supplementary Table 6 Values attributed to each gene considering the four prioritization parameters: density in the Hopfield network; number of GOs related to cancer development; number of patients with the gene active only in the tumor sample (biomarker); and node degree.

Supplementary Table 7 Characterization of each DEG identified in the RNA-Seq data set from NCBI Gene Expression Omnibus with respect to biological processes involved in the trastuzumab treatment response.
